# Supercritical Impregnation of *Mangifera indica* Leaves Extracts into Porous Conductive PLGA-PEDOT Scaffolds

**DOI:** 10.3390/polym16010133

**Published:** 2023-12-30

**Authors:** Diego Valor, Ignacio García-Casas, Antonio Montes, Ella Danese, Clara Pereyra, Enrique Martínez de la Ossa

**Affiliations:** 1 Department of Chemical Engineering and Food Technology, Faculty of Sciences, International Excellence Agrifood Campus (CeiA3), University of Cádiz, 11510 Puerto Real, Spain; diego.valor@uca.es (D.V.); ignacio.casas@uca.es (I.G.-C.); clara.pereyra@uca.es (C.P.); enrique.martinezdelaossa@uca.es (E.M.d.l.O.); 2 Faculty of Chemistry and Technology, University of Split, 21000 Split, Croatia; elladanese1@gmail.com

**Keywords:** PLGA, *Mangifera indica*, scCO_2_, foaming, impregnation, PEDOT

## Abstract

Plant leaves, such as those from *Mangifera indica*, represent a potential utilization of waste due to their richness in bioactive compounds. Supercritical CO_2_ allows these compounds to be incorporated into various matrices by impregnation. Combined with its ability to generate polymeric scaffolds, it represents an attractive strategy for the production of biomedical devices. For this purpose, conjugated polymeric scaffolds of biodegradable PLGA (poly(lactic-co-glycolic acid)) and PEDOT:PSS (poly(3,4-ethylenedioxythiophene)-poly(styrenesulfonate)), generated in situ by foaming, were employed for the supercritical impregnation of ethanolic mango leaves extract (MLE) in tissue engineering as a potential application. The extraction of MLE was performed by Enhanced Solvent Extraction. The effects of pressure (120–300 bar), temperature (35–55 °C), and depressurization rate (1–50 bar/min) on the physical/conductive properties and the impregnation of MLE were studied. The scaffolds have been characterized by liquid displacement, scanning electron microscope, resistance to conductivity techniques, measurements of impregnated load, antioxidant capacity and antimicrobial activity. Porosity values ranging 9–46% and conductivity values between 10^−4^–10^−5^ S/cm were obtained. High pressures, low temperatures and rapid depressurization favored the impregnation of bioactive compounds. Scaffolds with remarkable antioxidant activity were obtained (75.2–87.3% oxidation inhibition), demonstrating the ability to inhibit *S. aureus* bacterial growth (60.1 to 71.4%).

## 1. Introduction

The use of mango leaves (*Mangifera indica*) and the study of their inherent properties have been the subject of constant interest and scientific research throughout history, with origins dating back to ancient civilizations that astutely recognized the medicinal and nutritional benefits of these leaves. This accumulated wisdom about mango leaves and their evolution in use over the centuries has led to an increased recognition of these leaves as a veritable repository of bioactive compounds. Specifically, flavonoids, terpenoids, polyphenols and other constituents found in mango leaves have been identified as intrinsic agents endowed with properties such as antioxidant, anti-inflammatory and antimicrobial properties, giving them substantial value as a source of beneficial phytochemicals for the human organism, capable of contributing to the prevention and treatment of various diseases [1].

The use of supercritical carbon dioxide (scCO_2_) to extract compounds from plants and impregnation of these compound in a wide range of matrices, offers many advantages and benefits over conventional extraction and impregnation methods. The scCO_2_ extraction method is considered a green and environmentally friendly process due to its non-toxic, non-flammable and readily available nature [2]. Focusing on *Mangifera indica* plants, scCO_2_ can be tuned to have different solvating power by adjusting temperature and pressure. This selective solubility allows the extraction of specific compounds while leaving unwanted components behind [3]. In addition, scCO_2_ leaves no solvent residues, ensuring that the extracted compounds are free from contamination and suitable for use in the food, pharmaceutical and cosmetic industries. The relatively low temperatures and pressures used in scCO_2_ extraction minimize thermal degradation and preserve the bioactivity of the extracted compounds. As for supercritical impregnation (SSI), which has been described in detail in several articles [4,5,6], it has been used to impregnate mango leaves extract into polyester textiles [7], PLA filaments [8] or PLA 3D-printed devices [9].

PLGA (poly(lactic-co-glycolic acid)) is a biodegradable and biocompatible polymer widely recognized for its benefits in various biomedical applications. Its biocompatibility and biodegradability make it suitable for medical applications, including drug delivery and tissue engineering. PLGA allows for controlled drug release, with tunable degradation rates based on its lactic acid to glycolic acid ratio. Its versatility in forming different structures such as microspheres, nanoparticles, films, and scaffolds enhances its applicability [10].

PEDOT (poly(3,4-ethylenedioxythiophene)) is a conductive polymer with a number of valuable properties for a wide range of applications. Its inherent conductivity makes it a desirable material for electronics, sensors, and energy storage devices. In addition, PEDOT can be easily doped to further enhance its electrical properties, making it adaptable to specific requirements [11]. The polymer’s biocompatibility has also made it suitable for biomedical applications, particularly in neural tissue engineering. Its flexibility, transparency, and stability further enhance its utility in various fields [12]. Due to its impressive conductivity and compatibility, PEDOT continues to be a promising polymer for advancing technologies in electronics and biomedicine.

Supercritical CO_2_, which was initially used for extraction purposes, has emerged as a tool for the formation of scaffolds. The supercritical foam process [13,14] and the impregnation of a wide range of polymers have been explained in the literature [2,15,16,17]. Overall, the utilization of scCO_2_ in conjunction with conjugated polymers has proven to be a viable and sustainable approach in scaffold fabrication for tissue engineering applications [18]. With its ability to provide precise control over scaffold characteristics, this innovative technique holds promise for the development of advanced scaffolds with improved properties for tissue regeneration [19].

The conjugation of the two polymers, PLGA (poly(lactic-co-glycolic acid)) and PEDOT (poly(3,4-ethylenedioxythiophene)), holds significant potential for several advanced applications. By combining their different properties, researchers aim to create hybrid materials that exploit the advantages of both polymers. Within these two biodegradable polymers of particular interest, there have been numerous studies on the formation of scaffolds using supercritical CO_2_. Milovanovic et al., [20] developed systems for thymol release using PLGA, and Alvarez et al., [15] fabricated microcellular scaffolds using high pressure fluids to reduce the working temperature. The use of PLGA in combination with other polymers is also of interest in the literature. The formation of PLGA/PCL [21], PLGA/β-TCP [22], PLLA-grafted bioglass/PLGA (g-BG/PLGA) [23] and PLGA/hydroxyapatite [24] have been studied and characterized. These mixed polymers were investigated for their use in tissue engineering and their influence on cell growth. We highlight the previous study by Montes et al., [25] where PLGA-PEDOT:PSS were investigated in terms of the effect of pressure, temperature and contact time on the porosity, mechanical properties and conductivity of the conjugated polymeric scaffolds. On the other hand, a very limited number of investigations with PEDOT polymer using scCO_2_ have been reported in the literature, e.g., CoS/MXene/PEDOT [26] composites or for the addition of platinum nanoparticles to PEDOT/C matrix [27]. For instance, the incorporation of PEDOT into PLGA-based scaffolds could introduce electrical conductivity, making them suitable for tissue engineering applications that require electrical stimulation to enhance tissue growth and regeneration [28]. Additionally, the biocompatibility of both polymers ensures that the resulting hybrid materials are well tolerated by the body, further expanding their potential use in biomedicine [29].

In this paper, the authors aim to combine the field of the formation of conjugated polymers with tissue engineering properties (PLGA-PEDOT:PSS) with their functionalization with natural extracts (ethanolic mango leaves extract), paving the way for future in vivo or in vitro studies to confirm their excellent potential.

## 2. Materials and Methods

### 2.1. Materials

PLGA (lactide:glycolide 75:25) (poly(lactic-co-glycolic acid)) with Mw 76,000–115,000 and PEDOT:PSS (poly(2,3-dihydrothieno-1,4-dioxin)-poly(styrenesulfonate)) with conductivity > 200 S/cm, DPPH (2,2-dipheny l-1-picrylhydrazyl), Folin–Ciocâlteu reagent (FCR) and Triphenyl Tetrazolium Chloride (TTC) were purchased from Sigma–Aldrich (Spain). Absolute ethanol was purchased from PanReac AppliChem (Barcelona, Spain). CO_2_ (99.8% purity) was supplied by Linde (Barcelona, Spain). *Mangifera indica* L. leaves (Kent) were collected by Finca Experimental ‘La Mayora’, Superior Centre of Scientific Researches, CSIC (Málaga, Spain).

### 2.2. Enhanced Solvent Extraction of Mango Leaves

The enhanced solvent extraction of *Mangifera indica* leaves (MLE) was carried out with adjustments to a previously established protocol [17]. For this purpose, 125 g of dried and crushed leaves were placed into a paper filter cartridge, which was then placed inside a 0.5 L high-pressure vessel provided by Thar Technologies (Pittsburgh, PA, USA, SF500). The high-pressure system was equipped with precise temperature control, two high-pressure pumps (one for CO_2_ and the other for the co-solvent), an automatic back-pressure regulator (BPR), and a cyclonic separator at the end of the system in order to separate the CO_2_ from the liquid.

The extractions were carried out in batch mode using a mixture of CO_2_ and ethanol (7% *v*/*v*) under controlled conditions of 200 bar and 80 °C, maintained for a period of 3 h. The mixture was pumped at a rate of 10 g/min. Upon completion, the final extract was stored at 4 °C and protected from light to ensure preservation of chemical composition and stability.

### 2.3. One-Step Supercritical Foaming and Impregnation Process

In order to generate scaffolds with an ordered porous structure, suitable mechanical and electrical properties for tissue engineering, coupled with bioactive properties provided by the compounds from *Mangifera indica* leaves extract, the processes of polymeric foaming and supercritical impregnation were carried out in a single step. The experiments were carried out in a RESS250 lab scale unit (Thar Technologies, Pittsburgh, PA, USA) described in detail in previous works [25]. This process represents an additional step compared to the work on the formation of conjugated scaffolds. This time, in addition to scaffold formation, the bioactive compounds are impregnated by depositing a natural extract in the impregnation chamber. The ultimate goal is to obtain scaffolds with similar physical properties but with powerful antioxidant and/or antimicrobial capabilities. Due to the equilibrium of interactions between the supercritical phase, the matrix and the active ingredient, the impregnation process facilitates the transport of bioactives into the polymeric matrix. This is typically achieved by sorption and swelling phenomena. Such interactions contribute to improved diffusion of the active into the matrix, making the impregnation process in many cases more efficient and faster than various conventional impregnation methods.

**Figure 1:**
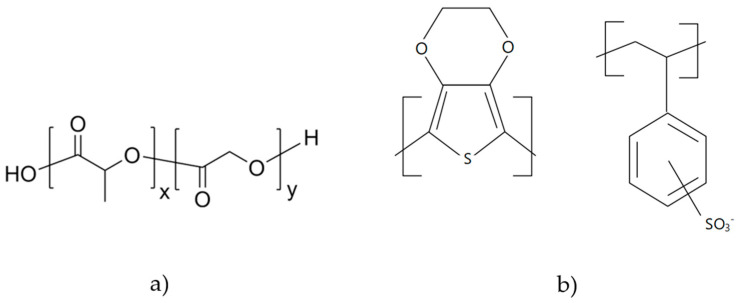
Chemical structure of the polymers used in the impregnation/foaming process, (**a**) PLGA (poly(lactic-co-glycolic acid)) and (**b**) PEDOT:PSS (poly(3,4-ethylenedioxythiophene)-poly(styrenesulfonate)).

The system features a 250 mL vessel where the temperature, pressure, and CO_2_ depressurization rate are monitored and controlled through software. In this case, the ethanolic extract to be impregnated into the polymer scaffold is placed at the bottom of the vessel to avoid direct contact with the polymers. For each experiment, 25 mg of PLGA 75:25 and 200 µL of PEDOT:PSS were combined and subsequently formed into a circular tablet (30 mm^3^) using a tablet press machine. The chemical structure of the used polymers is shown in Figure 1. Additionally, 6 mL of ethanolic MLE at a concentration of 60 mg/mL was placed at the bottom of the vessel. The polymer mixture was allowed to interact with the CO_2_ for the prescribed time, allowing the CO_2_ to penetrate the polymer to achieve the desired plasticizing effect. Controlled pressure release was then applied at the specified rate via the automatic back pressure regulator valve, resulting in the polymeric foaming and impregnation of antioxidants compounds into the final polymer structure in a single step.

Experiments (mixed level factorial 3 · 2^2^) were carried out to determine the most suitable conditions for the impregnation of MLE into the scaffolds and to evaluate the effect of the most important variables in such processes. The process was conducted under various pressures (120–300 bar), temperatures (35–55 °C), and depressurization rates (1–50 bar/min) to assess their influence on the morphological characteristics, the % of impregnation of the extract and the activity in terms of antibacterial growth and antioxidant power of the generated scaffolds. The fixed parameters utilized, the studied variables and responses can be observed in Table 1. The response variables included the conductivity of the samples, the impregnated load, and the porosity. A total of 14 experiments were performed in duplicate, with 2 center points.

### 2.4. Scanning Electron Microscopy

The structure and pore size of the foamed scaffolds were analyzed using the scanning electron microscope Nova NanoSEM 450TM (Elecmi, Zaragoza, Spain) at an accelerating voltage of 2.0 kV. In order to enhance the sample conductivity and achieve superior image quality during SEM analysis, a thin layer of gold (10 nm) was sputtered onto each sample’s cross-section before examination. The ImageJ software (version 1.54f), provided by the National Institutes of Health, USA was employed to process the SEM images.

### 2.5. Expansion Degree (%EXP)

The change in sample volume after supercritical CO_2_ treatment was quantified by determining the final expansion degree (%EXP). The initial volume (V_0_) of each tablet was measured directly according to its uniform surface and shape. The final volume (V) was then determined by immersing the scaffolds in ethanol and using the liquid displacement method. The expansion factor was calculated using the following expression:

(1) $$\%\;\mathrm{EXP}=\frac{V-V_{0}}{V}\times 100$$

### 2.6. Estimated Porosity

The porosity assessment of the foamed scaffolds was conducted using the liquid displacement method. Ethanol was chosen as the preferred working fluid due to its ability to penetrate the polymeric structure without inducing any disruption. The estimated porosity calculation involved comparing the void volume (representing the pores) with the total volume of the scaffolds, following the principles of Archimedes’ principle [30,31]. The porosity percentage was determined using the following equation:

(2) $$\mathrm{Porosity}\;(\%)=\frac{\left(V_{1}-V_{3}\right)}{\left(V_{2}-V_{3}\right)}\times 100$$

Here, V_1_ refers to the initial volume of ethanol, V_2_ represents the volume with the sample fully immersed until saturation, and V_3_ indicates the residual volume of ethanol after removing the sample. This method facilitated estimation of the scaffolds’ porosity, providing crucial insights into their internal structure.

### 2.7. Electrical Conductivity Measurement

The method described in previous works [25,32] with minor modifications was used to determine the conductivity of the different samples. The electrical conductivity of the PLGA-PEDOT-MLE scaffolds was measured using a resistance meter (TRMS Fluke 87-V) by the two-probe method. The size of the cut pieces for each test was 10 mm^2^. A voltage range of 0 to 10 V was used to measure the resistance to the passage of current, assisted by two copper sheets (200 μm). This measurement was carried out in triplicate and the results are expressed in terms of conductivity (S/cm).

### 2.8. Impregnation Load of the Extract

To quantify the loaded content of mango leaf extract within the scaffolds, a UV-Vis spectrophotometry method was employed, utilizing a Synergy™ HTX Multi-Modal Microplate Reader (Izasa, Madrid, Spain). To achieve this objective, 30 mg of each impregnated scaffold was completely degraded using 2.5 mL of dimethyl sulfoxide, which is capable of dissolving the PLGA with the lactide:glycolide ratio employed. Following this, the absorbance of the solutions was measured at a detection wavelength of 318 nm. This wavelength corresponds to the highest absorbance of MLE, coinciding with the maximum absorbance of mangiferin, without interference from the utilized polymers. To determine the final MLE impregnation percentage, a calibration curve was constructed in dimethyl sulfoxide, within the range of 0.2–4.0 mg/mL. The impregnated load was determined using the following equation:

(3) $$\%\;\mathrm{MLE}\;\mathrm{impregnated}=\frac{mMLE}{mPolymers}\times 100$$

where mMLE is the amount of ethanolic MLE impregnated into the polymers and mPolymers is the amount of polymer. The impregnation loadings were determined in duplicate.

### 2.9. Antioxidant Activity

The antioxidant potential of polymeric scaffolds impregnated with bioactive compounds from mango leaves was determined using the DPPH free radical spectroscopy method, following a procedure reported in a previous contribution with slight modifications [33]. Firstly, at different concentrations, the antioxidant capacity of the extract used was determined using a 6 × 10^−5^ M DPPH ethanolic solution. Subsequently, these concentrations of MLE (0.1 mL) were combined with 3.9 mL of DPPH solution and allowed to react for 3 h in the absence of light. Following this incubation period, absorbance at 515 nm was determined using a Synergy™ HTX Multi-Modal Microplate Reader (Izasa, Madrid, Spain). Following this, the oxidation inhibition percentage (%OI) was determined using Equation (4)

(4) $$\%\mathrm{OI}=\frac{A_{0}-A_{i}}{A_{0}}\times 100$$

where A_0_ represents the absorbance at the initial time and A_i_ represents the absorbance after 3 h.

In the case of impregnated scaffolds, 10 mg of each were immersed in the same DPPH solution (4 mL), and the absorbance was measured at 515 nm after 8 h, allowing the diffusion of scaffold compounds into the solution. The percentage of inhibition (%OI) for the impregnated scaffolds was calculated using Equation (4). All experiments were performed in duplicate.

### 2.10. Determination of the Antibacterial Activity of Impregnated Scaffolds

The antimicrobial activity of the impregnated PLGA-PEDOT scaffolds against gram-positive bacteria (*S. aureus*) was examined for the samples with the best impregnation results. In each antibacterial test, samples were sterilized by UV light and then 10 mg of impregnated scaffolds were used and placed in sterilized glass tubes (13 mm × 100 mm) together with LB broth medium and left for 24 h at 37 °C, to facilitate the potential release of impregnated polyphenols. Subsequent to this step, the samples were inoculated in order to achieve the targeted bacterial concentration of 1.5 × 10^6^ CFU/mL and were incubated for 24 h at 37 °C. After this, 200 µL of each tube was added to a multi-plate being treated with 20 µL of TTC solution (0.5% *w*/*v*). The plate was then incubated at 37 °C for 30 min. The red formazan solution obtained, which is indicative of cell activity and viability, was assessed by spectrophotometry at a wavelength of 500 nm. The percentage of bacterial growth inhibition was then calculated using the following equation:

$$\mathrm{Bacterial}\;\mathrm{growth}\;\mathrm{inhibition}\;(\%)=1-\frac{C_{i}}{C_{0}}\times 100$$

where C_i_ is the cell concentration and C_0_ is the cell concentration in the medium without impregnated scaffolds (negative control). To estimate the real effect of the impregnated samples against bacterial growth, controls without scaffolds and with non-impregnated scaffolds were also tested. Tests were done in triplicate.

## 3. Results and Discussion

### 3.1. Characterization of the Impregnated Scaffolds

**Figure 2:**
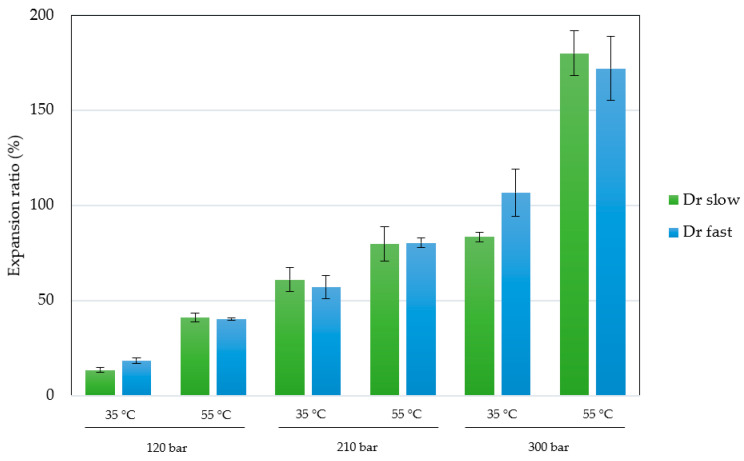
Effects of parameters shown via a Pareto chart for expansion ratio on polymeric scaffolds at different conditions of pressure, temperature and depressurization rate (Dr).

**Figure 3:**
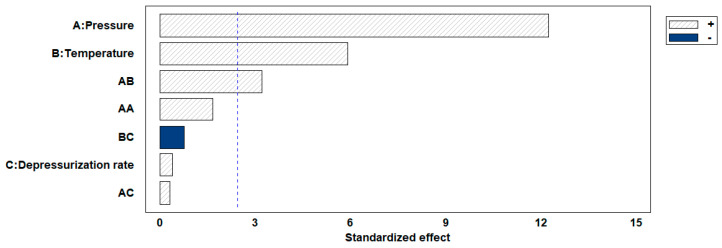
Pareto chart for expansion ratio response. The blue color indicates a negative effect of each variable, while shading indicates a positive effect.

**Figure 4:**
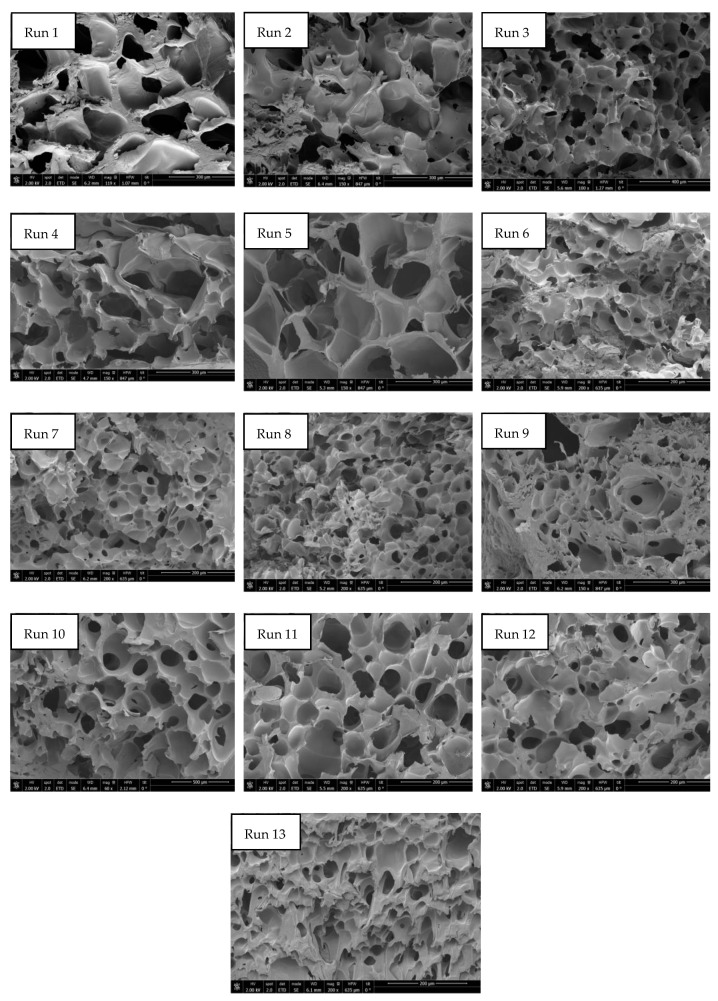
SEM images of formed scaffolds at different conditions of pressure, temperature and depressurization rate (Runs 1–13). Scale bar of runs 1,2,4,5 and 9 = 300 µm; run 3 = 400 µm; runs 6,7,8,11 and 12 = 200 µm; run 10 = 500 µm.

The permanent change in the final volume (expressed as expansion ratio) of the scaffolds after the foaming/impregnation process due to polymer swelling was studied for the various variables. The results obtained are shown in Figure 2. The expansion values range from 13.3% to 18.3% for experiments conducted at 120 bar, up to expansions of 180.2% when 300 bar, 55 °C and slow decompression were applied. Only pressure (*p*-value 0.0004), temperature (*p*-value 0.0010) and their binary interaction (*p*-value 0.0185) had a statistically significant effect on polymer expansion. To visualize these effects more clearly, the Pareto diagram for the expansion factor is presented in Figure 3. The chart plots a reference line to indicate which effects are statistically significant to the study. Pressure had a positive effect, causing a significant increase in expansion when increased from 120 to 300 bar. Increased pressure allows more CO_2_ to be absorbed by the polymer matrix and also decreases the nucleation energy barrier [30], leading to more CO_2_ bubbles in the interior and increased pores and porosity in the final scaffold. Equivalent behaviour is observed in the literature on pressure rise in terms of porosity and expansion [34,35]. The expansion factor proved to be highly dependent on porosity rate so that it followed the same trend as can be observed in the estimated porosity (%) results presented in Table 2. In addition, the internal morphology of the scaffolds, the formed pores and their distribution are shown in Figure 4. In the majority of SEM images, a large number of pores can be observed within the scaffold, as it is a cross-sectional image. The distinction between PLGA and PEDOT is not visible to the naked eye, as is common in such processes, due to their dispersion throughout the scaffold, as confirmed in the previous study [25] that motivated this research. A more organized pore structure is seen in experiments performed at higher pressure, possibly due to the increased ability of CO_2_ to penetrate between the polymer chains with less hindrance under these conditions.

In relation to temperature, when experiments were carried out at a higher temperature (55 °C), higher expansion factors and consequently higher percentages of porosity were obtained. When temperatures rise the glass transition of the polymer, in this case the main polymer PLGA 75:25 has a glass transition temperature (Tg) of 45–47 °C [36], the polymer achieves its rubbery state and CO_2_ will diffuse readily into the polymer matrix, resulting in increased volume growth upon depressurization phase. 

Increasing the temperature has the potential to alter the structural characteristics of PLGA-PEDOT and consequently its transport properties. Beyond the glass transition temperature (Tg), the increased chain mobility of the polymer may allow for improved diffusion of the supercritical phase, allowing for improved sorption of more CO_2_, thereby enhancing the swelling-foam process.

Several studies in the literature have investigated the fabrication of porous PLGA scaffolds using alternative techniques, including 3D printing, electrospinning and electrospun methods [37,38] or more conventional fabrication techniques such as salt leaching, gas forming, phase separation, and freeze-drying. Alternatively, supercritical foaming produced macropores in the range of 150–500 µm, exhibiting a wider distribution of porosities and allowing precise control of these parameters with minor variations in key parameters. Due to the wide range of potential applications for these polymers, and their versatility and additional properties, there is a wide range of activities in which they can be used. Considering the expansion factor and porosity obtained in the range of 10–46% with the one-step foaming/impregnation process, the obtained scaffolds, when compared to other polymers used in the literature, would be viable for use in cell infiltration [39], chondrogenesis [40], osteogenesis [41] or smooth muscle cell differentiation [42].

The development of scaffolds for engineered tissues has advanced with the incorporation of conductive materials to enhance scaffold bioactivity. These materials exploit different properties that have been shown to be beneficial in tissue engineering applications and combine them in anticipation of enhanced cellular responses to stimulation, whether mechanical or electrical. The integration of conductive materials into these bioactive synthetic scaffolds has the potential to improve regeneration outcomes by mitigating current factors that limit the effectiveness of existing scaffold materials [43]. Regarding the measured conductivity levels presented in Table 1, an inverse relationship is evident—the higher the expansion factor, the lower the conductivity. The reduced conductivity of the scaffolds can be attributed to the heightened porosity, leading to an increased presence of trapped air, and a greater disorder among the PEDOT:PSS molecules [44]. The conductive scaffolds developed in this study exhibit valuable electrical characteristics, attaining a maximum conductivity of 3.78 × 10^−4^ S/cm in run 4. Consequently, these products could emerge as promising candidates for applications in nerve, bone, vascular, or cardiac muscle tissue replacements, where the ability to conduct electrical signals is crucial [45].

### 3.2. Extract Loading (%MLE)

**Figure 5:**
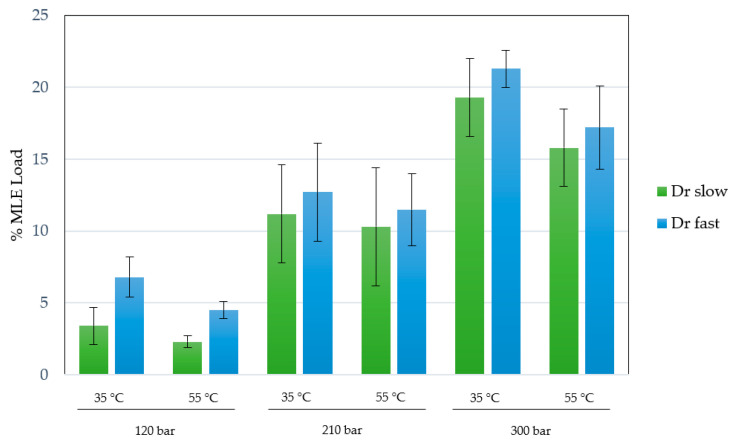
%MLE loading on impregnated scaffolds at 120–300 bar, 35–55 °C and 1–50 bar/min depressurization rate. All experiments were performed in duplicate.

An ethanolic mango leaves extract obtained through an enhanced extraction technique was utilized, as per prior contributions [17]. In this case, the MLE concentration was 60 mg/mL of ethanolic solution, with an extraction yield of 18.3% at 200 bar, 80 °C after 3 h. The %MLE loading in different experiments was measured and the effect of pressure, temperature and depressurization rate was evaluated. The results obtained under the different conditions are shown in Figure 5.

**Figure 6:**
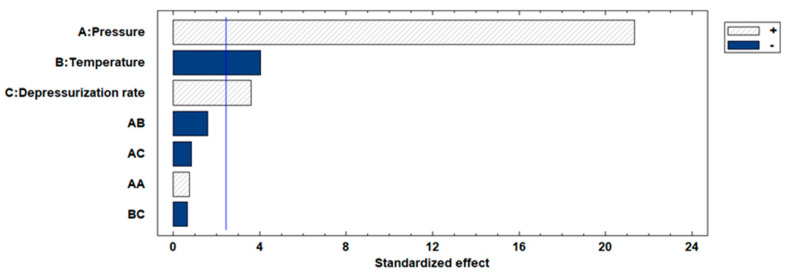
Effects of parameters shown via Pareto chart for %MLE loading. The blue color indicates a negative effect of each variable, while shading indicates a positive effect.

The impregnation percentages of the extract were obtained within a range of 2.4–21.3%. Studying the influence or statistical significance of each of the factors examined in the process (pressure, temperature, or depressurization ratio), all of them demonstrated a significant effect on the impregnation percentage of the extract in the produced scaffolds. Figure 6 shows the Pareto chart of the studied variables on the %MLE loading. On the other hand, binary interactions did not exhibit significant effects. With respect to individual factors, the effect was positive for pressure (*p*-value 0.0011) and depressurization ratio (*p*-value 0.0114), whereas temperature (*p*-value 0.0069) showed an opposite effect with a confidence level of 95%. The most favorable conditions (among those studied in the present work) to obtain a high %MLE load impregnation were 300 bar, 35 °C and a fast depressurization rate.

The mechanism that primarily determines the incorporation of the extract is the affinity between the polymeric material, in this case the PLGA-PEDOT blend, and the active agents. The study of potential interactions between the components of the extract and the polymer is challenging due to the complex nature of the extract composition. However, the functionalities of the primary constituents are taken into account in the analysis. The main compounds identified in the ethanolic extracts of mango leaves obtained by supercritical CO_2_ extraction are mangiferin and various derivatives of quercetin and iriflophenones [46]. For these compounds to be incorporated into the polymer by supercritical impregnation, significant chemical interactions must occur, causing molecular dispersion within the polymer. The main compounds in the extract have in common the presence of multiple hydroxyl groups, which can potentially form hydrogen bonds with the carbonyl groups present in the PLGA chains [20,47] or with the SO_3_H groups of PEDOT:PSS [48]. Nevertheless, not only hydrogen bonds will be formed due to the multitude of compounds present in such compositions, but the high degree of hydrophilic character in these polyphenols may potentially shift the partition coefficient toward the polymer rather than the supercritical phase.

The effects of the individual studied variables were significant for the impregnated load of the extract. In the case of pressure, it is evident that a higher impregnation of bioactive compounds was achieved using higher pressures. The increase in pressure leads to two phenomena that can contribute to increasing the impregnation percentage in this type of process. On one hand, a change in the concentration gradient of polyphenols present in the extract between the supercritical phase and the polymer results in differences in the final impregnation. The solubility of these compounds in CO_2_ increases with pressure, leading to saturation (as the extract was in excess concentration in the impregnation chamber). This creates a concentration gradient of the compounds to be impregnated, promoting their incorporation into the polymeric matrix. This phenomenon, with a positive effect of pressure, is reported in numerous studies on supercritical impregnation in polymers [49,50,51]. In addition, with a simultaneous foaming process, the polymer swelling phenomenon, which increases with higher pressure, can facilitate the incorporation of compounds during impregnation as diffusion through the polymer is enhanced. The strong effect of pressure observed in the experiments carried out could also be explained in this way [52].

With regard to the negative effect of temperature, and considering, as mentioned above, that the experiments were carried out under supersaturation conditions with an excess of extract, in these cases, an increase in temperature leads to a decrease in the solubility of CO_2_, resulting in a reduction in dissolved compounds. This should explain the overall decrease in %MLE observed when the process is carried out at 55 °C. Similar cases have been documented in the literature with this negative temperature effect, such as impregnating caffeic acid in PET/PP films [53] or thymol in cellulose acetate films [54], among others. However, an increase in temperature could lead to a structural change in the polymer, increasing the diffusion phenomena of the supercritical phase, as observed with increasing pressure. However, in this case, the solubility of the extract in CO_2_ seems to be the dominant process for MLE loading.

With regard to the influence of the depressurization rate, it was observed that, while maintaining pressure and temperature constant, a generally higher impregnation of compounds is achieved with rapid depressurization (50 bar/min). This can be explained by the greater affinity of the compounds in the extract towards the supercritical phase than the PLGA-PEDOT, resulting in the preference of these compounds to remain in CO_2_. Slow depressurization may favor the release of these compounds along with CO_2_ to the exterior.

Concerning the influence of the rate of depressurization, it was observed that, while maintaining the pressure and temperature constant, a generally higher impregnation of compounds is obtained with rapid depressurization (50 bar/min). This phenomenon can be elucidated by the greater affinity of the compounds in the extract for the supercritical phase than the PLGA-PEDOT, resulting in a preference for these compounds to remain in the CO_2_, hence, a slow depressurization may favor the release of these compounds along with CO_2_ to the exterior [51].

In this way, the optimal conditions were selected based on the highest degree of impregnation of the MLE, with parameters set at 300 bar, 35 °C, and rapid depressurization.

### 3.3. Antioxidant Activity of Impregnated Scaffolds

**Figure 7:**
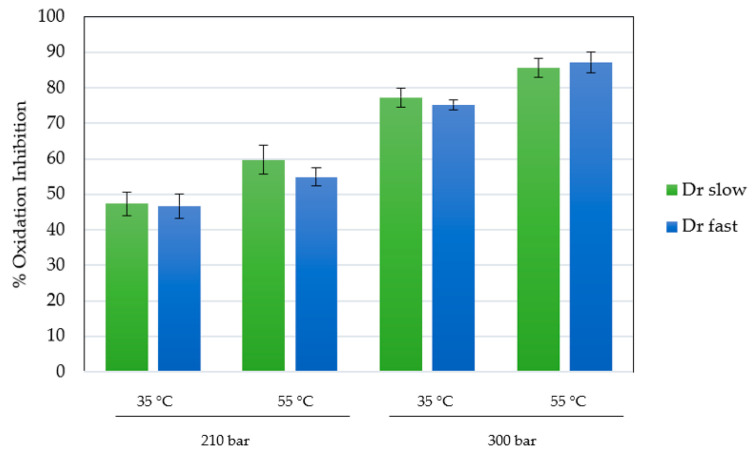
Percentages of oxidation inhibition for MLE loaded polymer scaffolds.

The antioxidant activity of the scaffolds impregnated under the best conditions (runs at 210 and 300 bar), with respect to the total impregnated load, was determined using the DPPH assay. The ability of the compounds presents in MLE to reduce DPPH and their capacity to reduce the presence of free radicals can be easily determined by the method tested. This capacity has been previously verified in the form of an extract and impregnated into other matrices, yielding high values of oxidation inhibition [17]. The presence of these free radicals or reactive oxygen species (ROS), can lead to various pathologies, inducing the well-known oxidative stress, potentially causing cardiovascular or neurodegenerative problems. The results for the scaffolds, expressed as percentage inhibition of oxidation, are shown in Figure 7. In order to compare the impregnation performance of the compounds and their activity, the extract was analyzed separately in liquid form. The used extract showed an isolated inhibition of DPPH oxidation of 92.2%.

In general, the results are consistent with those obtained in the total impregnated load, consistently achieving higher inhibition in the scaffolds produced at higher pressure (75.2–87.3%), being the variable that predominantly controls the process of impregnation-foaming in his work. Under similar conditions, in previous studies with the same type of extract in calcium alginate, inhibitions close to 80–90% were achieved at 300 bar in batch mode [17]. Cejudo and colleagues achieved a 45% oxidation inhibition by impregnating 3% mango ethanolic extract into PLA filaments at 400 bar (the highest pressure studied) and 55 °C [55]. The difference from the higher data in the present study confirms the complexity of the equilibrium reached under supercritical conditions between the compounds and the chosen impregnation matrix. In this case, the highly porous structure of PLGA in the form of a scaffold allows for better diffusion of the impregnated compounds into the medium. Of particular note is the consistent observation that antioxidant activity was higher in experiments conducted at elevated temperatures. These minor differences could be attributed to changes in structural properties and, consequently, transport properties induced by the increase in temperatures above the glass transition temperature.

### 3.4. Determination of the Antibacterial Activity of Impregnated Scaffolds

The investigation of the antibacterial properties of the impregnated PLGA-PEDOT scaffolds against the gram-positive bacteria *S. aureus* was carried out in vitro using the tetrazolium/formazan method. When exposed to bacteria, TTC is reduced, resulting in the formation of red formazan, a quantity directly proportional to the number of viable active cells. Results of antimicrobial activity were quantified as the percentage inhibition of cell growth after 24 h, using standard bacterial growth (negative control) as a baseline for comparison. The selected samples for analysis were those with higher extract loads and greater antioxidant activity, all produced at a pressure of 300 bar. The obtained results are presented in Table 3 expressed as percentage inhibition of *S. aureus* bacterial growth. The activity of the non-impregnated scaffolds was also analyzed to observe the real contribution of the impregnated compounds. As can be seen in Table 2, the non-impregnated scaffold (produced at 300 bar, 55 °C and slow depressurization) showed antimicrobial activity, which was enhanced in all cases where the bioactive compounds of interest were impregnated. The antimicrobial activity of conductive polymers is well documented in the literature, where an antibacterial agent with a cationic charge penetrates the membranes of bacterial cells, causing disruptions in membrane integrity that culminate in the death of the bacteria [56,57].

The impregnated scaffolds produced through the foaming/supercritical impregnation process exhibited antibacterial activity, with bacterial growth inhibition values ranging from 60.1 to 71.4%. Stronger bacterial inhibition was observed in scaffolds with higher antioxidant activity, particularly those produced at 300 bar and 55 °C. The antimicrobial activity of the scaffolds can mostly be attributed to compounds derived from *Mangifera indica*, including phytochemicals such as tannins, alkaloids, flavonoids or polyphenols. This may also extend to antiviral or antifungal activities. The mechanisms involved in this inhibitory activity of mango leaves’ extract compounds can be diverse, including changes in the properties of the bacterial cell membrane, the formation of a complex with iron required by microbes, the disruption of microbial enzyme synthesis or the interference with the replication cycle of viruses, among others [58]. In the literature, numerous studies showcase the development of scaffolds utilizing antibiotics, carbon nanomaterials, or peptides, among other agents, targeted for tissue engineering and regenerative medicine with a high antibacterial capacity [59,60]. The antibacterial activity results, coupled with the values and wide range of porosities, conductivity and high antioxidant activity, suggest that scaffolds produced by the proposed one-step foaming/impregnation method are viable for potential use in bone, cardiovascular, osteogenesis or neural tissue engineering.

## 4. Conclusions

The supercritical impregnation of *Mangifera indica* leaves extract proved successful when conducted in combination with the foaming process of PLGA/PEDOT:PSS scaffolds. The effect of some key process variables was investigated. Pressure and temperature had a significant positive effect on the foaming process, increasing the expansion and porosity of the scaffolds to a maximum of 46%. Conductive scaffolds were obtained by the addition of PEDOT, with conductivities between 10^−4^ and 10^−5^ S/cm, inversely related to the scaffold expansion.

Higher pressures and low temperatures favored the MLE loading by increasing the concentration gradient in excess and the diffusivity of CO_2_ within the polymer matrix. The optimal conditions for impregnation of the MLE extract were 300 bar, 35 °C and a depressurization rate of 50 bar/min. Scaffolds with higher extract loadings were tested for antioxidant and antibacterial activity. They showed high inhibition of oxidation (75.2–87.3%) with significant potential against pathologies induced by oxidative stress. Also, the scaffolds tested were able to inhibit the growth of *S. aureus* by up to 71.4%.

The outcomes from antibacterial/antioxidant activity, combined with the diverse porosity and conductivity values indicate the viability of employing scaffolds created through the suggested single-step foaming/impregnation technique in potential applications such as bone, cardiovascular, osteogenesis, or neural tissue engineering. However, further investigation is essential to assess the mechanism of antioxidant release in simulated physiological media. In addition, in vitro and in vivo assays are essential to ensure cell viability performance.

## Figures and Tables

**Table 1:** Design of experiments for foaming and impregnation process. The fixed parameters and different levels of studied variables are shown.

Fixed Parameters		Studied Variables	
Contact time	2 h	Pressure (bar)	120, 210, 300
Pressurization rate	10 g min^−1^	Temperature (°C)	35, 55
MLE volume	10 mL	Depressurization rate (bar/min)	1, 50
Polymer tablet size	30 mm^3^		

**Table 2:** Design of experiments for foaming/impregnation of PLGA-PEDOT:PSS with *Mangifera indica* extract. The variables used for the different runs are shown, where P: Pressure, T: Temperature and Dr: Depressurization ratio.

Experiments				Studied Responses	
Runs	P (bar)	T (°C)	Dr (bar/min)	Conductivity (S/cm)	Porosity (%)
1	120	35	1	2.71 × 10^−4^	9.3 ± 0.1
2	120	35	50	1.65 × 10^−4^	10.3 ± 0.4
3	120	55	1	1.35 × 10^−4^	15.8 ± 1.2
4	120	55	50	3.78 × 10^−4^	12.4 ± 1.1
5	210	35	1	1.43 × 10^−4^	17.9 ± 0.9
6	210	35	50	4.28 × 10^−5^	19.5 ± 1.3
7	210	55	1	6.54 × 10^−5^	26.8 ± 2.4
8	210	55	50	1.21 × 10^−5^	26.8 ± 1.8
9	300	35	1	2.47 × 10^−5^	37.4 ± 3.4
10	300	35	50	1.26 × 10^−5^	39.2 ± 0.2
11	300	55	1	3.48 × 10^−5^	46.1 ± 2.6
12	300	55	50	5.71 × 10^−5^	42.1 ± 1.2
Center point	210	45	25.5	1.71 × 10^−5^	35.6 ± 4.2
Center point	210	45	25.5	1.24 × 10^−5^	33.8 ± 3.2

**Table 3:** Percentage of inhibition of bacterial growth for experiments with higher impregnation load and antioxidant activity and non-impregnated scaffold.

Run			
P (bar)	T (°C)	Dr (bar/min)	% Bacterial Growth Inhibition
300	35	1	60.1 ± 3.1
300	35	50	62.6 ± 6.1
300	55	1	71.4 ± 2.3
300	55	50	69.2 ± 3.1
Non-impregnated scaffold			21.7 ± 6.7

## Data Availability

Data are contained within the article.

## References

[1] Rymbai H., Srivastav M., Sharma R.R., Patel C.R., Singh A.K. (2013). Bio-active compounds in mango (*Mangifera indica* L.) and their roles in human health and plant defence—A review. J. Hortic. Sci. Biotechnol..

[2] Meneses M.A., Caputo G., Scognamiglio M., Reverchon E., Adami R. (2015). Antioxidant phenolic compounds recovery from *Mangifera indica* L. by-products by supercritical antisolvent extraction. J. Food Eng..

[3] Villacís-Chiriboga J., Voorspoels S., Uyttebroek M., Ruales J., Van Camp J., Vera E., Elst K. (2021). Supercritical CO_2_ extraction of bioactive compounds from mango (*Mangifera indica* L.) peel and pulp. Foods.

[4] Banchero M., Mohamed S.S.Y., Leone F., Lopez F., Ronchetti S., Manna L., Onida B. (2019). Supercritical solvent impregnation of different drugs in mesoporous nanostructured zno. Pharmaceutics.

[5] Braga M.E.M., Costa V.P., Pereira M.J.T., Fiadeiro P.T., Gomes A.P.A.R., Duarte C.M.M., De Sousa H.C. (2011). Effects of operational conditions on the supercritical solvent impregnation of acetazolamide in Balafilcon A commercial contact lenses. Int. J. Pharm..

[6] Costa V.P., Braga M.E.M., Guerra J.P., Duarte A.R.C., Duarte C.M.M., Leite E.O.B., Gil M.H., de Sousa H.C. (2010). Development of therapeutic contact lenses using a supercritical solvent impregnation method. J. Supercrit. Fluids.

[7] Sanchez-Sanchez J., Fernández-Ponce M.T., Casas L., Mantell C., de la Ossa E.J.M. (2017). Impregnation of mango leaf extract into a polyester textile using supercritical carbon dioxide. J. Supercrit. Fluids.

[8] Rosales J.M., Cejudo C., Verano L., Casas L., Mantell C., Martínez de la Ossa E.J. (2021). Supercritical impregnation of pla filaments with mango leaf extract to manufacture functionalized biomedical devices by 3d printing. Polymers.

[9] Grosso P., Cejudo C., Sánchez-Gomar I., Durán-Ruiz M.C., Moreno-Luna R., Casas L., Pereyra C., Mantell C. (2022). Supercritical Impregnation of Mango Leaf Extract into PLA 3D-Printed Devices and Evaluation of Their Biocompatibility with Endothelial Cell Cultures. Polymers.

[10] Pan Z., Ding J. (2012). Poly(lactide-co-glycolide) porous scaffolds for tissue engineering and regenerative medicine. Interface Focus.

[11] Wichiansee W., Sirivat A. (2009). Electrorheological properties of poly(dimethylsiloxane) and poly(3,4-ethylenedioxy thiophene)/poly(stylene sulfonic acid)/ethylene glycol blends. Mater. Sci. Eng. C.

[12] Lari A., Sun T., Sultana N. (2016). PEDOT:PSS-Containing Nanohydroxyapatite/Chitosan Conductive Bionanocomposite Scaffold: Fabrication and Evaluation. J. Nanomater..

[13] Tai H., Mather M.L., Howard D., Wang W., White L.J., Crowe J.A., Morgan S.P., Chandra A., Williams D.J., Howdle S.M. (2007). Control of pore size and structure of tissue engineering scaffolds produced by supercritical fluid processing. Eur. Cells Mater..

[14] Jacobs L.J.M., Kemmere M.F., Keurentjes J.T.F. (2008). Sustainable polymer foaming using high pressure carbon dioxide: A review on fundamentals, processes and applications. Green Chem..

[15] Álvarez I., Gutiérrez C., Rodríguez J.F., De Lucas A., García M.T. (2020). Production of drug-releasing biodegradable microporous scaffold impregnated with gemcitabine using a CO_2_ foaming process. J. CO_2_ Util..

[16] Cabezas L.I., Fernández V., Mazarro R., Gracia I., De Lucas A., Rodríguez J.F. (2012). Production of biodegradable porous scaffolds impregnated with indomethacin in supercritical CO_2_. J. Supercrit. Fluids.

[17] Valor D., Montes A., García-Casas I., Pereyra C., Martínez de la Ossa E.J. (2021). Supercritical solvent impregnation of alginate wound dressings with mango leaves extract. J. Supercrit. Fluids.

[18] Barry J.J.A., Gidda H.S., Scotchford C.A., Howdle S.M. (2004). Porous methacrylate scaffolds: Supercritical fluid fabrication and in vitro chondrocyte responses. Biomaterials.

[19] Reverchon E., Cardea S., Rapuano C. (2008). A new supercritical fluid-based process to produce scaffolds for tissue replacement. J. Supercrit. Fluids.

[20] Milovanovic S., Markovic D., Mrakovic A., Kuska R., Zizovic I., Frerich S., Ivanovic J. (2019). Supercritical CO_2_—Assisted production of PLA and PLGA foams for controlled thymol release. Mater. Sci. Eng. C.

[21] Song C., Li S., Zhang J., Xi Z., Lu E., Zhao L., Cen L. (2019). Controllable fabrication of porous PLGA/PCL bilayer membrane for GTR using supercritical carbon dioxide foaming. Appl. Surf. Sci..

[22] Yang C., Kang Y.Q., Liao X.M., Yao Y.D., Huang Z.B., Yin G.F. (2010). Preparation of PLGA/β-TCP composite scaffolds with supercritical CO_2_ foaming technique. Front. Mater. Sci. China.

[23] Dong S., Wang L., Li Q., Chen X., Liu S., Zhou Y. (2017). Poly(L-lactide)-grafted bioglass/poly(lactide-co-glycolide) scaffolds with supercritical CO_2_ foaming reprocessing for bone tissue engineering. Chem. Res. Chinese Univ..

[24] Xin X., Guan Y., Yao S. (2018). Bi-/multi-modal pore formation of PLGA/hydroxyapatite composite scaffolds by heterogeneous nucleation in supercritical CO_2_ foaming. Chinese J. Chem. Eng..

[25] Montes A., Valor D., Penabad Y., Domínguez M., Pereyra C., de la Ossa E.M. (2023). Formation of PLGA–PEDOT: PSS Conductive Scaffolds by Supercritical Foaming. Materials.

[26] Chetana S., Upadhyay S., Joshi N.C., Kumar N., Choudhary P., Sharma N., Thakur V.N. (2023). A facile supercritical fluid synthesis of cobalt sulfide integrated with MXene and PANI/PEDOT nanocomposites as electrode material for supercapacitor applications. FlatChem.

[27] Bozkurt G., Memioğlu F., Bayrakçeken A. (2014). Pt nanoparticles over PEDOT/carbon composites prepared by supercritical carbon dioxide deposition. Appl. Surf. Sci..

[28] Yu R., Zhang H., Guo B. (2022). Conductive Biomaterials as Bioactive Wound Dressing for Wound Healing and Skin Tissue Engineering.

[29] Zhang Q., Esrafilzadeh D., Crook J.M., Kapsa R., Stewart E.M., Tomaskovic-Crook E., Wallace G.G., Huang X.F. (2017). Electrical stimulation using conductive polymer polypyrrole counters reduced neurite outgrowth of primary prefrontal cortical neurons from NRG1-KO and DISC1-LI mice. Sci. Rep..

[30] Mou Z.L., Zhao L.J., Zhang Q.A., Zhang J., Zhang Z.Q. (2011). Preparation of porous PLGA/HA/collagen scaffolds with supercritical CO_2_ and application in osteoblast cell culture. J. Supercrit. Fluids.

[31] Moghadam M.Z., Hassanajili S., Esmaeilzadeh F., Ayatollahi M., Ahmadi M. (2017). Formation of porous HPCL/LPCL/HA scaffolds with supercritical CO_2_ gas foaming method. J. Mech. Behav. Biomed. Mater..

[32] Pooshidani Y., Zoghi N., Rajabi M., Haghbin Nazarpak M., Hassannejad Z. (2021). Fabrication and evaluation of porous and conductive nanofibrous scaffolds for nerve tissue engineering. J. Mater. Sci. Mater. Med..

[33] Valor D., Montes A., Calderón-Domínguez M., Aghziel I., Sánchez-Gomar I., Alcalá M., Durán-Ruiz M.C., Pereyra C. (2023). Generation of Highly Antioxidant Submicron Particles from Myrtus communis Leaf Extract by Supercritical Antisolvent Extraction Process. Antioxidants.

[34] Arora K.A., Lesser A.J., McCarthy T.J. (1998). Preparation and characterization of microcellular polystyrene foams processed in supercritical carbon dioxide. Macromolecules.

[35] Goel S.K., Beckman E.J. (1994). Generation of microcellular polymeric foams using supercritical carbon dioxide. I: Effect of pressure and temperature on nucleation. Polym. Eng. Sci..

[36] Carmagnola I., Nardo T., Gentile P., Tonda-Turo C., Mattu C., Cabodi S., Defilippi P., Chiono V. (2015). Poly(lactic acid)-based blends with tailored physicochemical properties for tissue engineering applications: A case study. Int. J. Polym. Mater. Polym. Biomater..

[37] Lai Y., Li Y., Cao H., Long J., Wang X., Li L., Li C., Jia Q., Teng B., Tang T. (2019). Osteogenic magnesium incorporated into PLGA/TCP porous scaffold by 3D printing for repairing challenging bone defect. Biomaterials.

[38] Yang W., Yang F., Wang Y., Both S.K., Jansen J.A. (2013). In vivo bone generation via the endochondral pathway on three-dimensional electrospun fibers. Acta Biomater..

[39] Rnjak-Kovacina J., Wise S.G., Li Z., Maitz P.K.M., Young C.J., Wang Y., Weiss A.S. (2011). Tailoring the porosity and pore size of electrospun synthetic human elastin scaffolds for dermal tissue engineering. Biomaterials.

[40] Kemppainen J.M., Hollister S.J. (2010). Differential effects of designed scaffold permeability on chondrogenesis by chondrocytes and bone marrow stromal cells. Biomaterials.

[41] Kasten P., Beyen I., Niemeyer P., Luginbühl R., Bohner M., Richter W. (2008). Porosity and pore size of β-tricalcium phosphate scaffold can influence protein production and osteogenic differentiation of human mesenchymal stem cells: An in vitro and in vivo study. Acta Biomater..

[42] Cho S.W., Kim I.K., Lim S.H., Kim D.I., Kang S.W., Kim S.H., Kim Y.H., Lee E.Y., Choi C.Y., Kim B.S. (2004). Smooth muscle-like tissues engineered with bone marrow stromal cells. Biomaterials.

[43] Marsudi M.A., Ariski R.T., Wibowo A., Cooper G., Barlian A., Rachmantyo R., Bartolo P.J.D.S. (2021). Conductive polymeric-based electroactive scaffolds for tissue engineering applications: Current progress and challenges from biomaterials and manufacturing perspectives. Int. J. Mol. Sci..

[44] Alegret N., Dominguez-Alfaro A., Mecerreyes D. (2019). 3D Scaffolds Based on Conductive Polymers for Biomedical Applications. Biomacromolecules.

[45] Zarei M., Samimi A., Khorram M., Abdi M.M., Golestaneh S.I. (2021). Fabrication and characterization of conductive polypyrrole/chitosan/collagen electrospun nanofiber scaffold for tissue engineering application. Int. J. Biol. Macromol..

[46] Fernández-Ponce M.T., Casas L., Mantell C., De La Ossa E.M. (2015). Use of high pressure techniques to produce *Mangifera indica* L. leaf extracts enriched in potent antioxidant phenolic compounds. Innov. Food Sci. Emerg. Technol..

[47] Pajnik J., Milovanovic S., Stojanovic D., Dimitrijevic-Brankovic S., Jankovic-Častvan I., Uskokovic P. (2022). Utilization of supercritical carbon dioxide for development of antibacterial surgical sutures. J. Supercrit. Fluids.

[48] Wang X., Feng G., Li M., Ge M. (2019). Effect of PEDOT:PSS content on structure and properties of PEDOT:PSS/poly(vinyl alcohol) composite fiber. Polym. Bull..

[49] Goñi M.L., Gañán N.A., Strumia M.C., Martini R.E. (2016). Eugenol-loaded LLDPE films with antioxidant activity by supercritical carbon dioxide impregnation. J. Supercrit. Fluids.

[50] Torres A., Romero J., Macan A., Guarda A., Galotto M.J. (2014). Near critical and supercritical impregnation and kinetic release of thymol in LLDPE films used for food packaging. J. Supercrit. Fluids.

[51] De Souza A.C., Dias A.M.A., Sousa H.C., Tadini C.C. (2014). Impregnation of cinnamaldehyde into cassava starch biocomposite films using supercritical fluid technology for the development of food active packaging. Carbohydr. Polym..

[52] Varona S., Rodríguez-Rojo S., Martín Á., Cocero M.J., Duarte C.M.M. (2011). Supercritical impregnation of lavandin (*Lavandula hybrida*) essential oil in modified starch. J. Supercrit. Fluids.

[53] Comin L.M., Temelli F., Saldaña M.D.A. (2012). Barley β-glucan aerogels as a carrier for flax oil via supercritical CO_2_. J. Food Eng..

[54] Milovanovic S., Stamenic M., Markovic D., Ivanovic J., Zizovic I. (2015). Supercritical impregnation of cellulose acetate with thymol. J. Supercrit. Fluids.

[55] Cejudo Bastante C., Casas Cardoso L., Fernández-Ponce M.T., Mantell Serrano C., Martínez de la Ossa E.J. (2019). Supercritical impregnation of olive leaf extract to obtain bioactive films effective in cherry tomato preservation. Food Packag. Shelf Life.

[56] Gupta S., Datt R., Mishra A., Tsoi W.C., Patra A., Bober P. (2022). Poly(3,4-ethylenedioxythiophene):Poly(styrene sulfonate) in antibacterial, tissue engineering and biosensors applications: Progress, challenges and perspectives. J. Appl. Polym. Sci..

[57] Nandhini P., Rajan M. (2022). Antimicrobial Activities of Conducting Polymers and Their Derivatives. Conducting Polymers.

[58] Alaiya M.A., Odeniyi M.A. (2023). Utilisation of Mangifera indica plant extracts and parts in antimicrobial formulations and as a pharmaceutical excipient: A review. Future J. Pharm. Sci..

[59] Hinderer S., Brauchle E., Schenke-layland K. (2015). Generation and Assessment of Functional Biomaterial Scaffolds for Applications in Cardiovascular Tissue Engineering and Regenerative Medicine. Adv. Healthc. Mater..

[60] Martin V., Ribeiro I.A., Alves M.M., Gonçalves L., Claudio R.A., Bettencourt A.F. (2019). Engineering a multifunctional 3D-printed PLA-collagen-minocycline-nanoHydroxyapatite scaffold with combined antimicrobial and osteogenic effects for bone regeneration. Mater. Sci. Eng. C.

